# Boron Modified Bifunctional Cu/SiO_2_ Catalysts with Enhanced Metal Dispersion and Surface Acid Sites for Selective Hydrogenation of Dimethyl Oxalate to Ethylene Glycol and Ethanol

**DOI:** 10.3390/nano11123236

**Published:** 2021-11-29

**Authors:** Deliang Yang, Runping Ye, Ling Lin, Rong Guo, Peiyu Zhao, Yanchao Yin, Wei Cheng, Wenpeng Yuan, Yuangen Yao

**Affiliations:** 1 Biological Engineering Technology Innovation Center of Shandong Province, Heze Branch of Qilu University of Technology (Shandong Academy of Sciences), Heze 274000, China; peiyuz0512@126.com (P.Z.); yycbm@163.com (Y.Y.); chengwei200300@163.com (W.C.); yuanwp@sdas.org (W.Y.); 2 Key Laboratory of Jiangxi Province for Environment and Energy Catalysis, Institute of Applied Chemistry, College of Chemistry, Nanchang University, Nanchang 330031, China; rye@ncu.edu.cn; 3 Key Laboratory of Coal to Ethylene Glycol and Its Related Technology, Fujian Institute of Research on the Structure of Matter, Chinese Academy of Sciences, Fuzhou 350002, China; llin@fjirsm.ac.cn (L.L.); rongg@fjirsm.ac.cn (R.G.)

**Keywords:** Cu/SiO_2_ catalyst, sol-gel method, boron promoter, dimethyl oxalate hydrogenation, ethylene glycol, ethanol

## Abstract

Boron (B) promoter modified Cu/SiO_2_ bifunctional catalysts were synthesized by sol-gel method and used to produce ethylene glycol (EG) and ethanol (EtOH) through efficient hydrogenation of dimethyl oxalate (DMO). Experimental results showed that boron promoter could significantly improve the catalytic performance by improving the structural characteristics of the Cu/SiO_2_ catalysts. The optimized 2B-Cu/SiO_2_ catalyst exhibited excellent low temperature catalytic activity and long-term stability, maintaining the average EG selectivity (Sel._EG_) of 95% at 190 °C, and maintaining the average EtOH selectivity (Sel._EtOH_) of 88% at 260 °C, with no decrease even after reaction of 150 h, respectively. Characterization results revealed that doping with boron promoter could significantly increase the copper dispersion, enhance the metal-support interaction, maintain suitable Cu^+^/(Cu^+^ + Cu^0^) ratio, and diminish metallic copper particles during the hydrogenation of DMO. Thus, this work introduced a bifunctional boron promoter, which could not only improve the copper dispersion, reduce the formation of bulk copper oxide, but also properly enhance the acidity of the sample surface, so that the Cu/SiO_2_ sample could exhibit superior EG selectivity at low temperature, as well as improving the EtOH selectivity at high temperature.

## 1. Introduction

**Scheme 1:**
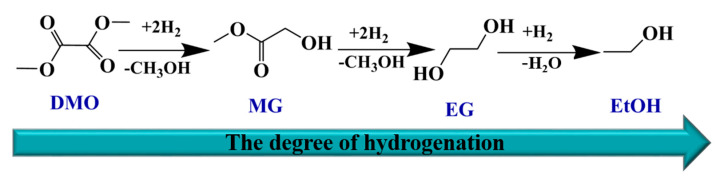
Hydrogenation reaction process of the DMO to MG, EG, and EtOH.

Ethylene glycol (EG) and ethanol (EtOH) are both versatile chemical raw materials and intermediates, which can be used for the synthesis of plentiful fine chemicals and applying in all walks of life [1,2,3]. The traditional preparation routes are derived from petroleum or biological routes. However, with the shrinkage of the crude oil resources and increasing environmental pollution, the coal chemical route based on C1 chemical technology has become a research hotspot in recent years [1,2,3]. Among them, the hydrogenation of DMO (dimethyl oxalate) is increasing important because its ability which can build a bridge between syngas and methyl glycolate (MG), EG and EtOH, etc. (Scheme 1). However, challenges still remain in how to obtain the corresponding hydrogenation target products according to market demand.

According to the literature, Cu-based catalysts are extensively investigated owing to the outstanding activity in C–O and C=O bonds and relative inactivity for C–C bonds in DMO hydrogenation [4,5,6]. Among them, the Cu/SiO_2_ catalysts presented good catalytic performance, low cost and environmentally friendly in DMO vapor-phase hydrogenation reaction [7,8,9]. However, pure Cu/SiO_2_ catalysts are difficult to meet the requirements of large-scale industrial applications owing to their poor activity and stability. Generally, the suitable Cu^+^/Cu^0^ ratio and the strong interactions between active ingredient copper and SiO_2_ support or promoter additives show a crucial influence over the catalytic performance of Cu/SiO_2_ catalysts. The deactivation of Cu/SiO_2_ catalysts is due to Cu coagulation and Cu^+^ species loss by valence transition during the reaction [10]. To solve this problem, numerous studies have been conducted on preparation methods and promoter additives [11,12,13,14,15,16,17,18,19,20,21,22]. In previous studies, different preparation methods, including sol–gel, ion–exchange, deposition–precipitation, ammonia evaporation hydrothermal, and ammonia evaporation method have been applied in preparing Cu/SiO_2_ catalysts with high metallic dispersion and moderate interaction between copper and silica [10,11]. Moreover, different kinds of species (e.g., Zn, Ni, B, In, Ag, or Mg, etc.) were doped to increase the Cu species dispersion and maintain a suitable Cu^+^/(Cu^+^ + Cu^0^) ratio, thereby improving the catalytic performance of the Cu/SiO_2_ catalyst [8,18,19,20,21]. Ye et al. have successfully synthesized β-cyclodextrin (β-CD) modified Cu-SiO_2_ catalysts by the sol-gel method, which presented the highest Sel._EG_ of 95.0% at 210 °C [22]. Chen et al. have synthesized mannitol modified Cu/SiO_2_ catalysts by ammonia evaporation method, which showed excellent catalytic activity (average Sel._EG_ of 95.0%) at 200 °C [23]. Ren et al. have introduced La promoter into Cu/SiO_2_ catalysts by hydrolysis precipitation method, which showed the outstanding catalytic performance with the Sel._EtOH_ of 95.8% at 250 °C [24]. However, there are still huge challenges need to be overcome, especially in terms of their poor low-temperature catalytic activity and stability.

As a promoter additive, B_2_O_3_ dopant can facilitate the formation of more surface defects and adjust acid-base properties of Cu/SiO_2_ catalysts. Yin et al. concluded that the content of boron promoter was crucially important to improve the Con._DMO_ and Sel._EG_ in the DMO hydrogenation. Appropriate addition of B_2_O_3_ by the ammonia-evaporation method could increase the dispersity and specific surface area of Cu species, thus improving the catalytic performance of Cu/SiO_2_ catalysts [9]. Zhu et al. found that the introduction of boron promoter to Cu/SiO_2_ catalysts by precipitation-gel method could effectively suppress the growth of metallic copper particles and increase the dispersion of Cu species, which could enhance catalytic activity and stability [25]. Zhao et al. found that the doping of boric acid on Cu/SiO_2_ catalyst by ammonia evaporation hydrothermal method had a crucial influence on its catalytic performance for the deep-hydrogenation of DMO to EtOH [26]. As we know, compared with other preparation methods, the Cu/SiO_2_ catalysts developed by the sol-gel method had the advantages of smaller copper particles, higher dispersion, and larger surface area. However, there were almost no reports on doping boron promoter to improve the catalytic performance of Cu/SiO_2_ catalysts by sol-gel method. Moreover, there were also relatively few reports on the effect of the boron promoter on the Cu/SiO_2_ catalysts for simultaneously catalyzing DMO hydrogenation reaction to produce EG and EtOH.

In our previous work, we had introduced cyclodextrin to modify the Cu/SiO_2_ catalysts by sol-gel method, but the cyclodextrin was neutral and did not exist in the sample after calcination, so it was difficult to change the surface acidity of the sample [22]. In this work, boron promoter modified Cu/SiO_2_ bifunctional catalysts for the efficient hydrogenation of DMO to EG and EtOH were synthesized by sol-gel method. By adjusting the content of B species and reaction temperature, the catalytic performance for the selective hydrogenation of DMO to EG and EtOH could be significantly improved. Variety of characterization methods were utilized to deeply investigate their structures, surface chemistry and structure-activity relationship. Hence, the optimized 2B-Cu/SiO_2_ catalyst is highly efficient, low-cost, environmentally friendly, as well as showing an industrial application prospect.

## 2. Materials and Methods

### 2.1. Catalyst Preparation

The 15 wt% Cu/SiO_2_ catalysts with different B/Cu molar ratio were developed by using sol-gel method. The specific details of the synthesis method were described as follows: (1) Under the circumstance of constant stirring, 13 g Cu(NO_3_)_2_·3H_2_O was added to a 300 mL beaker with 45 g deionized water. (2) A certain amount of H_3_BO_3_ (B/Cu molar ratio of 0.25, 1, 2, and 3) were dissolved in the above blue transparent solution. And then 100 g of ethanol and 70 g of tetraethylorthosilicate (TEOS) were added and stirred vigorously in a water bath for 1–2 h at 70 °C, forming a blue jelly-like sol-gel. (3) The blue jelly-like sol-gel was changed into the blocky B-Cu/SiO_2_ catalyst precursors by aging for 24 h at room temperature and drying for 61 h (17 h at 70 °C, 40 h at 90 °C, and then 4 h at 120 °C). (4) Finally, after calcining (5 h at 300 °C), crushing and sieving to 20–40 meshes, the B-Cu/SiO_2_ catalyst precursors was then denoted as *x*B-Cu/SiO_2_ catalysts (*x* representing B/Cu molar ratio).

### 2.2. Catalyst Characterization

To deeply investigate their structures, surface chemistry and structure-activity relationship of boron modified Cu/SiO_2_ catalysts, variety of characterization (such as BET, N_2_O titration, XRD, H_2_-TPR, NH_3_-TPD, TEM, STEM-EDX mapping, XPS, XAES, etc.) methods were utilized in this work. More operational details about the characterization methods were presented in the supporting information.

### 2.3. Catalytic Reaction

As presented in Figure S1, all the *x*B-Cu/SiO_2_ catalysts for the hydrogenation of DMO to EG and EtOH were evaluated in a fixed-bed reactor with a 120 mm stainless steel single-tube (10 mm internal diameter). Briefly, 10 mL of catalyst (20–40 meshes) was located in the constant temperature zone of the reaction tube, and the remaining space of the tube was filled with the silica sand. All the *x*B-Cu/SiO_2_ catalysts were reduced under 99.99% hydrogen for 8 h at 350 °C and 1.0 MPa pressure prior to the evaluation. The reactants (20 wt% DMO methanol solution) were injected into the gasification chamber and mixed with hydrogen (99.99%) by using high-pressure pump, after naturally cooling down to the reaction temperature. The reaction conditions were as follow: the DMO weight liquid hourly space velocity (LHSV) = 0.2 h^−1^, H_2_/DMO molar ratio = 50, P = 2.0 MPa. After the reaction ran smoothly for 6 h, the reactants and products were separated and analyzed by a gas chromatograph (GC-900C) equipped with FID detector. The conversion rate of DMO and the selectivity to various products (MG, EG and EtOH) are calculated as follows:

$$\mathrm{Conversion}\;(\mathrm{DMO})=\frac{n_{\mathrm{DMO},\mathrm{in}}\;-\;n_{\mathrm{DMO},\mathrm{out}}}{n_{\mathrm{DMO},\mathrm{in}}}\times 100\%$$

$$\mathrm{Selectivity}\;(\mathrm{MG})=\frac{n_{\mathrm{MG}}}{n_{\mathrm{DMO},\mathrm{in}}\;-\;n_{\mathrm{DMO},\mathrm{out}}}\times 100\%$$

$$\mathrm{Selectivity}\;(\mathrm{EG})=\frac{n_{\mathrm{EG}}}{n_{\mathrm{DMO},\mathrm{in}}\;-\;n_{\mathrm{DMO},\mathrm{out}}}\times 100\%$$

$$\mathrm{Selectivity}\;(\mathrm{EtOH})=\frac{n_{\mathrm{EtOH}}}{n_{\mathrm{DMO},\mathrm{in}}\;-\;n_{\mathrm{DMO},\mathrm{out}}}\times 100\%$$

## 3. Results and Discussion

### 3.1. Structural and Textural Properties of the xB-Cu/SiO_2_ Catalysts

The physicochemical properties of the *x*B-Cu/SiO_2_ catalysts were summarized in Table 1. Notably, all the actual loading of the copper and boron measured by ICP-OES were slightly lower than the theoretical values, but the measured B/Cu ratios were basically consistent with the theoretical values. As presented in Figure S2, all the *x*B-Cu/SiO_2_ catalysts showed typical IV type isotherms with H_2_-type hysteresis loops, indicating that all the catalysts possessed mesoporous structure [27].

Besides, Table 1 also showed the, pore volume (*V*_p_) and pore diameter (*D*_p_) and BET surface area (S_BET_) of the *x*B-Cu/SiO_2_ catalysts. Obviously, the introduction of B species slightly influenced the *V*_p_ and *D*_p_ of the *x*B-Cu/SiO_2_ catalysts. In addition, the S_BET_ of the *x*B-Cu/SiO_2_ catalysts displayed a volcanic change with the increase of boron content, and reached its summit in case of B/Cu molar ratio at 2, with the maximum value of 449.3 m^2^·g^−1^. However, further increasing the content of B species would lead to a gradual decline in SBET, which could be due to the addition of excessive boron promoter covered the surface of the *x*B-Cu/SiO_2_ catalysts [25]. 

As we know, the Cu dispersion (D_Cu_) and Cu surface area (S_Cu_) are considered to be two key elements that determine the catalytic activity of the Cu/SiO_2_ catalysts in the ester hydrogenation reactions [28]. As shown in Table 1, after introducing B element to Cu/SiO_2_ catalyst, D_Cu_ and S_Cu_ have been improved to a certain extent. It was worth noting that 2B-Cu/SiO_2_ possessed the highest D_Cu_ (21.6%) and S_Cu_ (23.8 m^2^·g^−1^), however further increasing the content of B species would lead to a gradual decline in both D_Cu_ and S_Cu_. This might be due to low boron loading acting as an isolating agent, inhibited thermal transmigration and agglomeration of Cu particles during the H_2_ reduction process. Excessive boron loading formed a thin film on the copper particles at the surface of the catalyst, which hindered the contact between the copper particles and N_2_O, thereby reducing D_Cu_ and S_Cu_ [25].

### 3.2. XRD and TEM

**Figure 1:**
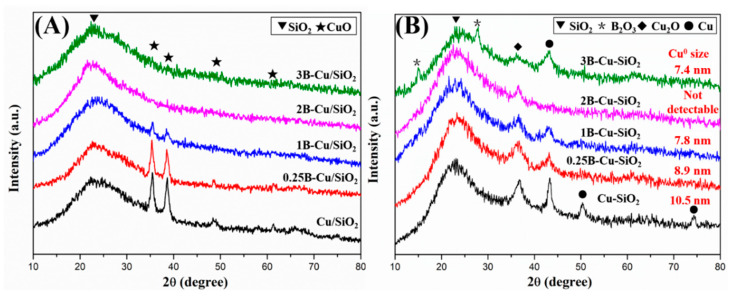
XRD patterns of the calcined (**A**) and reduced (**B**) *x*B-Cu/SiO_2_ catalysts.

To study the effect of boron promoter on the phase structure of Cu/SiO_2_ catalyst, the *x*B-Cu/SiO_2_ catalysts were characterized by XRD patterns. Figure 1A showed the XRD characterization patterns of calcined *x*B-Cu/SiO_2_ catalysts. Notably, the crystal structure of *x*B-Cu/SiO_2_ catalysts did not changed significantly after doping with boron promoter, and an amorphous SiO_2_ diffraction characteristic peak emerged at 2θ = 22.8°. When the B/Cu molar ratio x was lower than 2, obvious CuO characteristic diffraction peaks could be found at 2θ = 35.5°, 38.7° and 48.8° (JCPDS 45-0937), and the intensity of the CuO diffraction peaks gradually decreased with the increase of boron content. With further increasing boron loading leaded to the disappearance of CuO diffraction peaks. These results confirmed that adding boron promotor could effectively inhibit the agglomeration and growth of CuO particles, and CuO particles were highly dispersed in the SiO_2_ support. Figure 1B showed the XRD characterization spectrum of the reduced *x*B-Cu/SiO_2_ catalysts. Upon reduction at 220 °C, CuO diffraction peaks (35.5°, 38.7° and 48.8°) of the *x*B-Cu/SiO_2_ catalysts disappeared, and three diffraction peaks at 2θ of 43.3°, 50.4°, and 74.1° emerged, which were assigned to the Cu (111), (200) and (220) faces (JCPDS 04-0836), respectively. Overall, the half-peak width of Cu^0^ diffraction peaks increased and their intensity gradually diminished with the increase the content of boron promoter. When the B/Cu molar ratio was 2, the intensity of Cu^0^ diffraction peaks became unobservable, indicating that the Cu particle size was too small to detect and the Cu particles were more uniformly dispersed. However, further increase boron loading could make the Cu^0^ diffraction peaks become sharper. Besides, the average copper particle sizes of the reduced *x*B-Cu/SiO_2_ catalysts calculated by Scherrer equation were also summarized in Figure 1B. Notably, the average copper particle sizes gradually decreased from 10.5 nm for Cu/SiO_2_ to 7.4 nm for 3B-Cu/SiO_2_ catalyst. These results suggested that doping with boron promoter could decrease the copper particle sizes and promote the D_Cu_. The 2B-Cu/SiO_2_ catalyst exhibited the smallest particle size and maximum dispersion, which could be attributed to the most suitable interaction between Cu and B species at the B/Cu molar of 2. On the contrary, the addition of too much or too little B species could lead to the growth of copper particles and the decrease of dispersion. Moreover, the B_2_O_3_ diffraction peaks emerged at 2θ = 15° and 27.8° (JCPDS 06-0297) when the B/Cu molar ratio was 3. In addition, the peak at 2θ of 36.5° was ascribable to the (111) lattice face of Cu_2_O (JCPDS05-0667). The formation of Cu_2_O species originated from the strong interaction between the copper and the silicon support, which made Cu^2+^ partially reduce to Cu^+^ [29].

**Figure 2:**
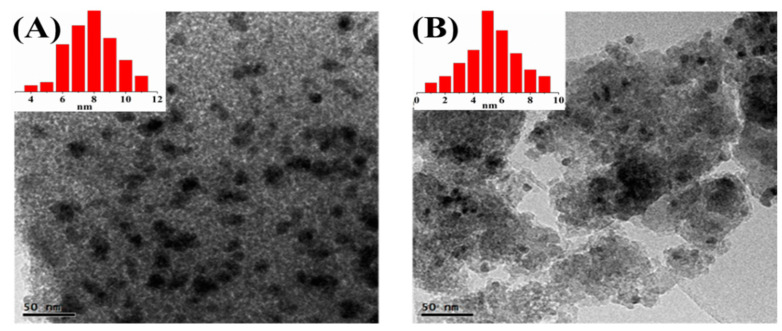
TEM images with the corresponding Cu particles size distribution diagrams of reduced (**A**) Cu/SiO_2_ and (**B**) 2B-Cu/SiO_2_ catalysts.

To further observe the surface morphology of the catalysts, the reduced Cu/SiO_2_ and 2B-Cu/SiO_2_ catalysts were characterized by TEM images, and the relevant results were shown in Figure 2. It was notable that the Cu particles on the surface of Cu/SiO_2_ catalyst were relatively large and agglomerate to a certain extent. The mean size of Cu particles of the Cu/SiO_2_ catalyst was about 8.4 nm based on the statistical results of TEM images. However, after adding an appropriate amount of boron promoter, the metallic Cu particles of the 2B-Cu/SiO_2_ catalyst were more uniformly dispersed without obvious agglomeration, and the average diameter was about 5.1 nm. Moreover, the enlarged TEM image and the EDS data in Figure S3 indicated that the Cu and B species were homogeneously evenly dispersed on the silica texture, overlapping with each other, implying that there was a strong interaction between active component Cu and boron promoter. The reason might be that Cu and B species were in close proximity, hindering the conversion of Cu^2+^ to Cu^0^ to a certain extent. The results of TEM images further proved that boron promoter could promote the D_Cu_ of the Cu/SiO_2_ catalysts by restraining the increase of Cu particles size, which were consistent with the conclusions of N_2_-adsorption, XRD and H_2_-TPR.

### 3.3. H_2_-TPR and NH_3_-TPD

**Figure 3:**
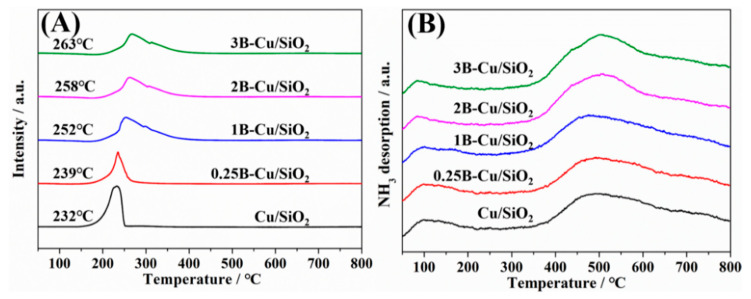
H_2_-TPR (**A**) and NH_3_-TPD (**B**) profiles of *x*B-Cu/SiO_2_ catalysts.

As presented in Figure 3A, all the calcined *x*B-Cu/SiO_2_ catalysts with different B/Cu ratios were systematically characterized by the H_2_-TPR technology, in order to reveal the influence of the boron loading on the reducibility of the catalysts. It was clearly found that there was a H_2_ consumption peak at about 232 °C of the unmodified Cu/SiO_2_ catalyst, which could be ascribed to the reduction of highly dispersed copper species [16]. After doping with boron promoter, the H_2_ consumption peak of the *x*B-Cu/SiO_2_ catalysts gradually shifted to the high temperature direction. These results could be attributed to the fact that B_2_O_3_ was an electrophilic substance, gaining electrons more easily than the supporter SiO_2_, and there was an intense interaction between the support, copper oxide, and boron oxide species [21]. With the increase of boron content, the copper species on the catalyst surface was more difficult to gain electrons. Moreover, all the total H_2_ consumption values calculated from the TPR results by taking CuO as standard material were lower than the total theoretical H_2_ consumption values (Table S1). These results suggested that Cu^+^ and Cu^0^ coexisted on the surface of the reduced catalysts, which was in concordance with the XRD and XPS results. 

In addition, a small shoulder peak appeared near the main reduction peak of the *x*B-Cu/SiO_2_ catalysts when the B/Cu molar ratio was higher than 1, which may be due to the formation of new copper species (such as copper borate). The results of H_2_-TPR further proved that the basic CuO and the acidic B_2_O_3_ had a strong interaction.

As illustrated in Figure 3B, the NH_3_-TPD technique was adopted to analyze the effect of boron promoter on the the strength and quantity of acid sites on the xB-Cu/SiO_2_ catalysts. It’s worth noting that all the xB-Cu/SiO_2_ catalysts presented two apparent NH_3_ desorption peaks (at about 105 °C and 500 °C), representing the weakly acidic and strong acidic sites, respectively [30]. The low-temperature and high-temperature peaks could be attributed to ammonia adsorbed on Si−OH and ammonia adsorbed on Cu particles, respectively [31]. As summarized in Table 2, the number of surface acidic sites increased to a certain extent with the increase of B/Cu molar ratio, indicating that the adding B species to Cu/SiO_2_ catalysts could increase the strength and quantity of surface acidic sites, which was consistent with previously reported results [9].

### 3.4. XPS and XAES 

**Figure 4:**
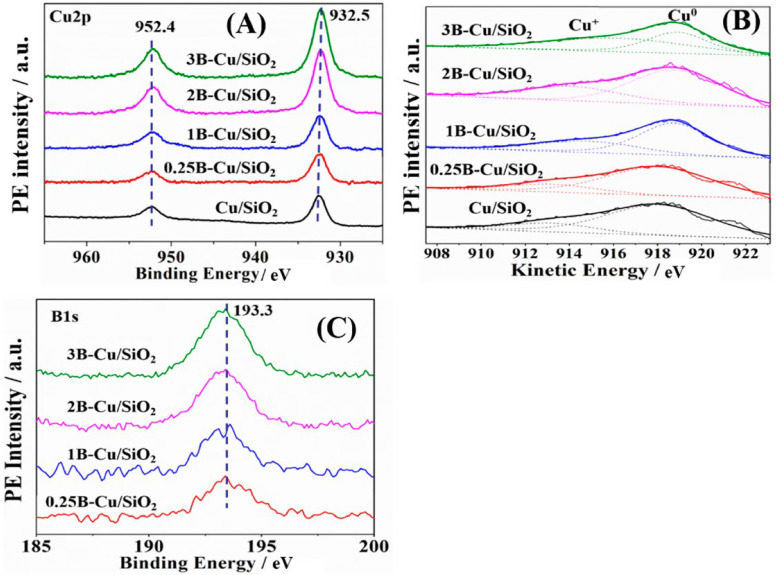
(**A**) Cu2p XPS spectra (**B**) Cu LMM XAES spectra (**C**) B1s XPS spectra of *x*B-Cu/SiO_2_ catalysts.

The XPS and X-ray induced Auger spectra (XAES) spectra of the reduced *x*B-Cu/SiO_2_ catalysts were shown in Figure 4, which were further measured to identify the surface composition and chemical state of catalysts. Remarkably, only two peaks were observed at binding energies of 932.5 eV and 952.4 eV belonging to Cu2p_3/2_ and Cu2p_1/2_, respectively (Figure 4A). This strongly suggested that the Cu^2+^ had been reduced to Cu^+^ or Cu^0^ because of the absence of 2p→3d satellite peaks around 933.5 eV or 934.9 eV, which was consistent with the XRD results. As presented in Figure 4C, the binding energy of B 1s peak center at 193.5 eV was attributed to trivalent boron (B^3+^), but no B^0^ peak was observed at 188.7 eV, which was corresponding to the result by Zhu et al. [25]. Notably, with the increase of boron content, the surface amount of B^3+^ gradually increased based on the intensity of B1s peaks of B1s XPS spectra.

Since the binding energies of Cu^+^ and Cu^0^ were located at approximately the same position, it was necessary to separate these two species by Cu LMM XAES spectrum. The asymmetric and broad Auger peak in the Cu LMM XAES spectrum could be deconvoluted into two overlapped peaks at 913.8–914.4 eV and 918.3–918.6 eV, representing Cu^+^ and Cu^0^ species, slightly higher than the value of the pure Cu/SiO_2_ catalyst [16,32]. The synergetic effect between Cu^+^ and Cu^0^ species on the surface of Cu-based catalysts was extensively acknowledged in the hydrogenation of DMO [5,6,11]. As summarized in Table 3, the surface Cu^+^/(Cu^+^ + Cu^0^) ratio was gradually increased from 59.5% to 69.2% with the increase of boron content, which corresponded with the results reported by Zhu et al. [25]. This might arise from the strong interaction between Cu species and B species, which resulted in a lower surface copper reduction degree and partial positive charge on the copper surfaces. Notably, the Cu^+^/(Cu^+^ + Cu^0^) ration of the optimal 2B-Cu/SiO_2_ catalyst (64.6%) was lower than that of 3B-Cu/SiO_2_ catalyst (69.2%), which could be attributed to the fact that the acidity and electron affinity of B_2_O_3_ were stronger than that of support silica, resulting in an increase in the amount of Cu^+^.

In addition, the Auger parameters (AP) of Cu^+^ and Cu^0^ species approximately the same as the previously published values of 1847.0 eV and 1851.0 eV, respectively [33].

### 3.5. Catalytic Performance Test

**Figure 5:**
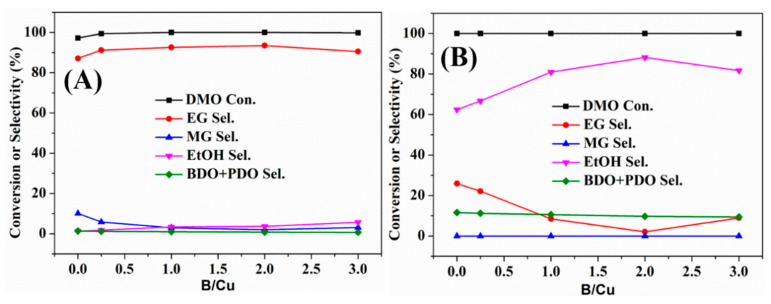
The correlation relationship between catalytic activity and B/Cu molar ratio of *x*B-Cu/SiO_2_ catalysts. Reaction conditions: LHSV = 0.2 h^−1^, P = 2.0 MPa, H_2_/DMO molar ratio = 50. (**A**) 200 °C and (**B**) 260 °C.

The gas-phase catalytic hydrogenation performance of the *x*B-Cu/SiO_2_ catalysts with different B/Cu molar ratios were investigated in a stainless steel fixed-bed reactor, and the related results were presented in Figure 5. Clearly, the DMO conversion (Con._DMO_) and EG selectivity (Sel._EG_) could be improved by introducing B element under identical conditions. Among them, the 2B-Cu/SiO_2_ catalyst with B/Cu molar ratio of 2 exhibited the highest Sel._EG_ of 93.5% (Con._DMO_ = 100%) at 200 °C. In addition, when the reaction temperature reached 260 °C, occurring more side reactions of excessive hydrogenation and dehydration. Notably, as the B/Cu molar ratio increased, the selectivity of EtOH (Sel._EtOH_) increased, whereas the selectivity of other byproducts including 1,2-butanediol (1,2-BDO) and 1,2-propanediol (1,2-PDO) decreased. Also, 2B-Cu/SiO_2_ catalyst with B/Cu molar ratio of 2 afforded the highest Sel._EtOH_ of 88.2% (Sel._Others_ = 9.7%) at 260 °C. This result might be interpreted by the fact of that doping boron promoter could increase the number of surface acidic sites, which facilitated the improvement of the selectivity of the EtOH and lowering the selectivity of 1,2-BDO and 1,2-PDO [31]. In short, higher or lower B/Cu molar ratio would lead to a decrease in Sel._EG_ or Sel._EtOH_ to a certain extent, indicating that adding boron promoter to Cu/SiO_2_ catalysts with suitable B/Cu molar ratio was more conducive to enhance the catalytic hydrogenation reaction activity of DMO hydrogenation to EG and EtOH. Remarkably, one of the most attractive features of the 2B-Cu/SiO_2_ catalyst was that we could produce EG and EtOH in the same reaction device by simply changing the reaction temperature.

**Figure 6:**
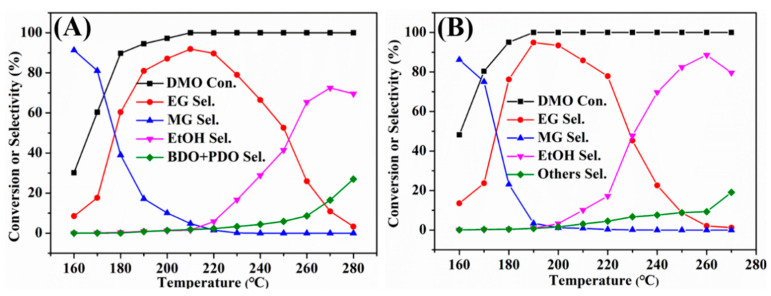
The correlation relationship between catalytic activity and reaction temperature of (**A**) Cu/SiO_2_ and (**B**) 2B-Cu/SiO_2_ catalysts.

The effects of different reaction temperatures on the catalytic performance of the Cu/SiO_2_ and 2B-Cu/SiO_2_ catalysts were explored and summarized in detail (Figure 6). The Con. _DMO_ and Sel._EG_ of the 2B-Cu/SiO_2_ catalyst sharply rose with the increase of temperature. And then the Sel._EG_ reached the maximum of 94.9% (Con._DMO_ = 99.99%) at 190 °C. However, under identical reaction conditions, the Con._DMO_ and Sel._EG_ of Cu/SiO_2_ catalyst were only 94.5% and 81.1%, respectively (Figure 6A). On further increasing the temperature, EG was then over-hydrogenated to produce EtOH, which leaded to a decrease in Sel._EG_ and an increase in Sel._EtOH_ of 2B-Cu/SiO_2_ catalyst. As a result, the Sel._EtOH_ reached the maximum of 88.2% (Sel._EG_ = 2.1%) at 260 °C, and then decreases to 79.1% (Sel._EG_ = 1.2%), which could be attributed to more side reactions occurring at higher temperature. However, under identical reaction conditions, the Sel._EtOH_ of Cu/SiO_2_ catalyst was only 65.4% (Sel._EG_ = 25.9%) at 260 °C. The conversion frequency (TOF) represented the intrinsic activity of the catalyst. As described in Table S2, the optimal 2B-Cu/SiO_2_ catalyst possessed the highest TOF value of 3.4 h^−1^, while the TOF of Cu/SiO_2_ catalyst was only 2.5 h^−1^. The results showed that the adding boron promoter to the catalyst could make the catalyst generating more active sites after reduction.

**Figure 7:**
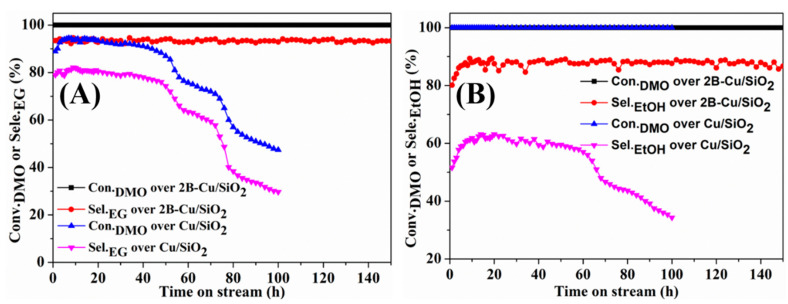
Long-term stability test of Cu/SiO_2_ and 2B-Cu/SiO_2_ catalysts. (**A**) 190 °C and (**B**) 260 °C.

Generally, the long-term stability of Cu-based catalysts was critical for the practical application for vapor-phase DMO hydrogenation. Hence, the long-term stability test of Cu/SiO_2_ and optimized 2B-Cu/SiO_2_ catalysts were evaluated under the same conditions. As shown in Figure 7A, the 2B-Cu/SiO_2_ catalyst exhibited excellent catalytic stability, which maintained its stable Con._DMO_ (100%) and Sel._EG_ (95%) at 190 °C, with no decrease even after 150 h of reaction. On the contrary, the Cu/SiO_2_ catalyst deactivated apparently within 100 h under identical reaction conditions. Both the Con._DMO_ and Sel._EG_ were gradually reduced, while MG gradually became the main product. When the reaction temperature was 260 °C, a similar trend could be observed in Figure 7B, where the Con._DMO_ and Sel._EtOH_ of 2B-Cu/SiO_2_ catalyst were maintained at about 100% and 88%, respectively. For the Cu/SiO_2_ catalyst, the Con._DMO_ was maintained around 100%, while the Sel._EtOH_ sharply decreased from 62% to 34% within 100 h under identical reaction conditions. Conversely, the selectivity of EG, 1,2-BDO and 1,2-PDO gradually rose. Besides, in order to better study the stability performance of the optimal 2B-Cu/SiO_2_ catalyst, the long-term stability was carried out under the condition of the initial DMO conversion rate of 100%. As presented in Figure S4, the 2B-Cu/SiO_2_ also excellent catalytic stability even after 50 h of reaction, maintaining its stable Con._DMO_ (80%) and Sel._EG_ (23.5%) at 170 °C. These results revealed that adding an appropriate amount of bifunctional boron promoter could significantly enhance the catalytic stability of Cu/SiO_2_ catalyst.

### 3.6. Structure–Performance Relationship 

**Figure 8:**
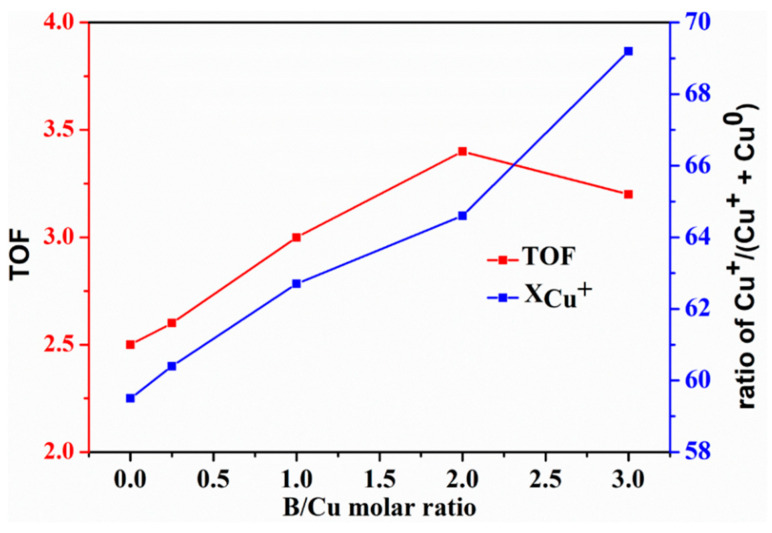
The correlation relationship between TOF and Cu^+^/(Cu^0^ + Cu^+^) intensity ratio.

**Figure 9:**
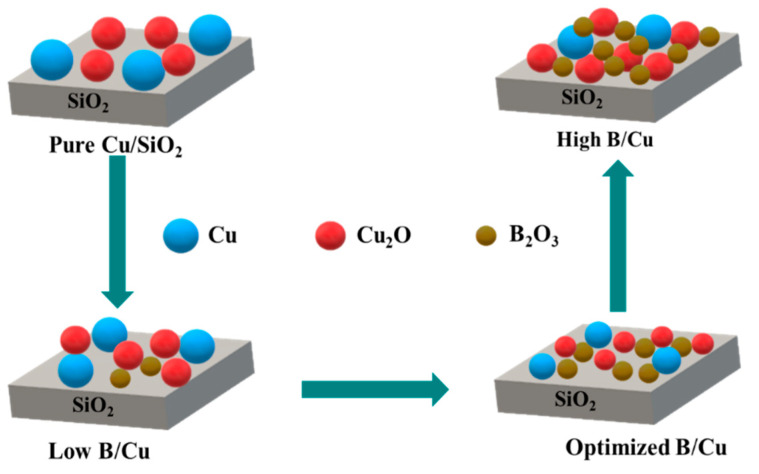
Schematic diagram of *x*B-Cu/SiO_2_ catalysts with the increase of B/Cu molar ratio.

As reported in previous work, Cu/SiO_2_ catalysts were extensively investigated in DMO hydrogenation reactions, and various promoters (e.g., Zn, Ni, In, Ag, or Mg, etc.) had been adopted to improve their catalytic performance [2,32,34,35,36,37]. Among them, doping with B species was one of the most useful methods to improve their performance by affecting D_Cu_. However, there were relatively few reports on the effect of the boron promoter on the Cu/SiO_2_ catalysts for simultaneously catalyzing DMO hydrogenation reaction to produce EG and EtOH. In our previous work, the cyclodextrin was introduced to modify the Cu/SiO_2_ catalyst by sol-gel method, but the cyclodextrin was neutral and did not exist in the sample after calcination, so it was difficult to change the acidity of the sample surface [22]. According to previous reports, appropriately reducing the basic sites and increasing the acid sites on the surface of Cu/SiO_2_ samples was beneficial to inhibit the conversion of ethanol to C3–C4OH, thereby improving the selectivity of EtOH at high temperatures [31,38]. Herein, the *x*B-Cu/SiO_2_ catalysts were synthesized by the sol-gel method with an appropriate B/Cu molar ratio could dramatically enhance the catalytic hydrogenation activity of Cu/SiO_2_ catalysts for DMO hydrogenation to EG and EtOH. The significantly alterations of Cu/SiO_2_ catalysts induced by boron promoter were found to be up to the Cu dispersion, reducibility, acidity–alkalinity, and distribution of Cu^+^ and Cu^0^ species, causing the corresponding alterations in catalytic performance. The XRD and TEM characterization results of the *x*B-Cu/SiO_2_ catalysts revealed that the addition of B species could inhibit the increase of Cu particles size and promote the dispersion of Cu species. The characterization results of EDS, H_2_-TPR, XPS, and XAES implied that the strong electronic interactions existed between the active component copper and boron promoter, affecting the distributions of copper species with different valence states. The synergetic effect between Cu^+^ and Cu^0^ species on the surface of Cu-based catalysts was extensively acknowledged in the hydrogenation of DMO [5,6,11,39,40,41,42,43,44,45,46]. In detail, the Cu^0^ sites dissociate and activate H_2_ molecule, and Cu^+^ sites adsorbed the carbonyl group. As shown in Figure 8, the TOF value and Cu^+^/(Cu^0^ + Cu^+^) intensity ratio presented a similar trend, increasing accordingly with the increase of B/Cu molar ratio (≤2). However, further increasing boron loading resulted in the opposite trend. The optimized 2B-Cu/SiO_2_ catalyst afforded the highest TOF value of 3.4 h^−1^, and the Cu^+^/(Cu^0^ + Cu^+^) intensity ratio reached the maximum of 69.2% when the B/Cu molar ratio was 3. These results could be attributed to the fact that the acidity and electron affinity of B_2_O_3_ were stronger than that of support silica, resulting in an increase in the amount of Cu^+^. However, excessive boron loading formed a thin film on the Cu particles on the surface of the catalyst, leading to a reduction in exposed and accessible Cu surface, which resulted in the decrease in catalytic activity. These results likewise demonstrate that an appropriate surface Cu^+^ and Cu^0^ concentration was one of the factors that affected the catalytic performance, but it only played a secondary role. The particle sizes, Cu dispersion, Cu surface area, and metal–support interaction, etc., also had significant effects on catalytic activity. Furthermore, Figure 9 presented a convincing schematic diagram of the *x*B-Cu/SiO_2_ catalysts with the increase of B/Cu molar ratio, based on the characterization results and previous reports.

**Figure 10:**
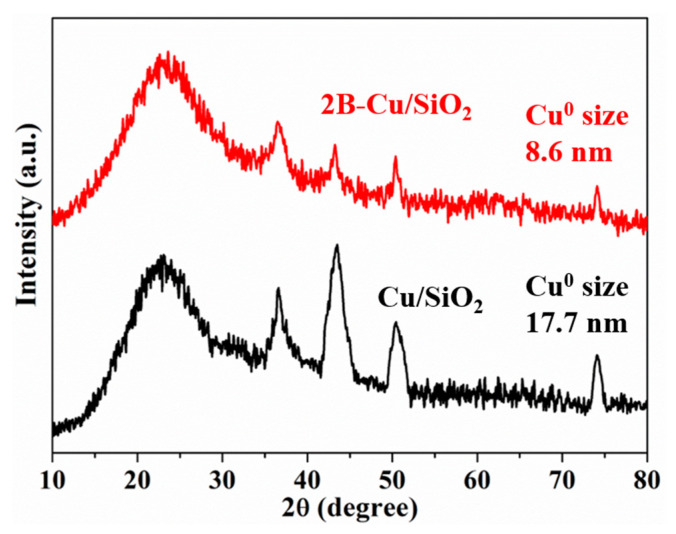
XRD patterns of Cu/SiO_2_ and 2B-Cu/SiO_2_ catalysts after 150 h long-term catalytic test.

**Figure 11:**
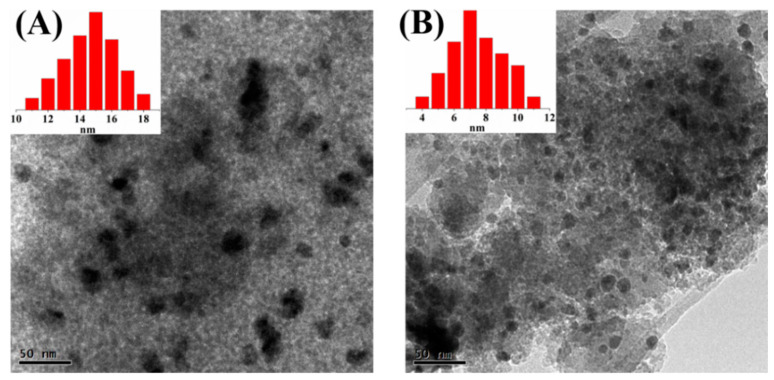
TEM images with the corresponding Cu particles size distribution diagrams of (**A**) Cu/SiO_2_ and (**B**) 2B-Cu/SiO_2_ catalysts after 150 h long-term catalytic test.

As we all know, the monometallic Cu/SiO_2_ catalysts showed poor catalytic stability in long-term DMO hydrogenation reactions. The influence of boron promoter on the stabilization of Cu/SiO_2_ catalysts had been extensively studied. Zhu et al. discovered that the introduction of B_2_O_3_ to Cu/SiO_2_ could greatly promote the dispersion of Cu species by inhibiting the growth of Cu particles, which could enhance catalytic stability [21]. He et al. reported that B-Cu/SiO_2_ catalysts exhibit excellent catalytic stability, which was mainly resulting from the strong interaction between B_2_O_3_ and surface Cu element, which maintained the suitable Cu^0^/Cu^+^ distribution and restrained the growth of Cu particles [24]. As shown in Table S3, the change in the D_Cu_ of 2B-Cu/SiO_2_ catalyst was basically negligible and only dropped from 21.6% to 20.4%. On the contrary, the D_Cu_ of Cu/SiO_2_ catalyst dramatically decreased from 17.9% to 12.5%, which resulted from the aggregation and growth of copper particles on catalyst surface. Calculated by on the Scherrer equation, the size of copper particles for the pure Cu/SiO_2_ catalyst agglomerated and increased to 17.7 nm from initial 10.5 nm after 150 h long-term stability test. Nevertheless, the size of copper particles for the optimal 2B-Cu/SiO_2_ catalyst only grew up to 8.6 nm under the identical condition (Figure 10). TEM characterization results of the optimal 2B-Cu/SiO_2_ and pure Cu/SiO_2_ catalysts after 150 h long-term stability test further indicated that the introduction of boron promoter could was conducive to suppressing the aggregation and promoting the dispersion of Cu species (Figure 11). Moreover, the Cu^+^/(Cu^0^ + Cu^+^) intensity ratio of the Cu/SiO_2_ catalyst was reduced from 59.5% to 48.9%, while that of the 2B-Cu/SiO_2_ catalyst changed slightly after long-term stable test (Table S3 and Figure S5). The result suggested that the destruction of the balance between the Cu^+^ and Cu^0^ activity sites could result in decrease in catalytic performance of Cu/SiO_2_ catalysts, which was consistent with previously reported results [11,18]. In conclusion, the main causes of Cu/SiO_2_ catalysts deactivation could be attributed to the aggregation and growth of copper particles and the decrease of surface Cu^+^/Cu^0^, and further proved that doping a suitable amount of B species into the Cu/SiO_2_ catalysts could effectively improve the stability of the catalyst. 

It is generally accepted that DMO hydrogenation reaction is a multi-step reaction. Firstly, DMO reacts with hydrogen to form MG, and then MG reacts with hydrogen to obtain EG. Finally, excessive hydrogenation of EG produces EtOH. Moreover, 1,2-BDO and 1,2-PDO may be derived from the dehydration reaction of EG with EtOH and methanol (MeOH), respectively. According to the previous reports, the acidic and basic sites on the surface of the catalyst are conducive to generate EtOH and C3−C4 diols (1,2-BDO and 1,2-PDO), respectively. From the NH_3_-TPD characterization results, the introduction of B species to *x*B-Cu/SiO_2_ catalysts could generate more acidic sites, thereby affecting the distribution of products, reducing the selectivity of 1,2-BDO and 1,2-PDO and improving the selectivity of EtOH at high temperature.

## 4. Conclusions

This work presents that the boron species promoted Cu/SiO_2_ bifunctional catalysts by sol-gel method exhibit remarkable catalytic activity and longtime stability for DMO hydrogenation to EG and EtOH. The target product of EG or EtOH could be regulated by facilely changing the reaction temperature. The optimal 2B-Cu/SiO_2_ catalyst exhibited the best low temperature catalytic activity and longtime stability among the *x*B-Cu/SiO_2_ catalysts. Adding the suitable B/Cu molar ratio was beneficial to reduce the size of copper particles, increase the Cu dispersion, enhance the metal–support interaction, regulate the surface copper species distribution, and then improve the synergetic effect of Cu^+^ and Cu^0^ species. Meanwhile, the introduction of boron promoter could enhance the surface acidity of the Cu/SiO_2_ catalysts to a certain extent, thereby improving the Sel._EtOH_ of the catalyst at high temperature. Besides, the long-term stability of 150 h test showed that the sintering of copper particles and reduction of the surface Cu^+^/Cu^0^ ratio were the major causes of deactivation of the Cu/SiO_2_ catalyst. However, we proved that doping a suitable amount of boron promoter into the pure Cu/SiO_2_ catalysts could restrain the increase of surface copper particles size, stabilize the surface Cu^+^/Cu^0^ ratio and effectively improve the stability of the catalyst. The current findings reflect that the bifunctional boron promoter may provide potential promising applications in other hydrogenation of carbon-oxygen bonds.

## Figures and Tables

**Table 1:** Textural properties and chemical compositions of *x*B-Cu/SiO_2_ catalysts.

Catalysts	Cu Loading (wt%) ^a^	B Loading (wt%) ^a^	Cu Dispersion (%) ^b^	S_Cu_ (m^2^·g^−1^) ^b^	S_BET_ (m^2^·g^−1^)	*V*_P_ (cm^3^·g^−1^) ^c^	*D*_p_ (nm) ^d^
Cu/SiO_2_	14.34	0	17.9	19.2	386.0	0.42	3.8
0.25B-Cu/SiO_2_	14.23	0.60	18.5	19.6	402.5	0.42	3.9
1B-Cu/SiO_2_	14.06	2.38	19.9	21.1	421.7	0.45	4.1
2B-Cu/SiO_2_	13.94	4.72	21.6	23.8	449.3	0.48	4.2
3B-Cu/SiO_2_	13.88	7.11	20.3	21.7	427.1	0.44	4.0

^a^ Determined by ICP-OES analysis. ^b^ Cu metallic surface area determined by N_2_O titration method. ^c^ Average pore volume determined by N_2_ adsorption–desorption isotherms. ^d^ Average pore diameter determined by N_2_ adsorption–desorption isotherms.

**Table 2:** Specific results of the NH_3_-TPD measurements over the *x*B-Cu/SiO_2_ catalysts.

Catalyst	Total NH_3_ Desorbed (μmol/g)	Density of Acidic Sites (μmol/m^2^)
Cu/SiO_2_ catalyst	76	0.20
0.25B-Cu/SiO_2_ catalyst	89	0.22
1B-Cu/SiO_2_ catalyst	102	0.24
2B-Cu/SiO_2_ catalyst	126	0.28
3B-Cu/SiO_2_ catalyst	137	0.32

**Table 3:** Surface Cu component of reduced catalysts based on Cu LMM deconvolution.

Catalyst	K.E. (eV) ^a^		A.P. (eV) ^b^		Cu2p_3/2_ B.E. (eV)	X_Cu_^+^ (%) ^c^
	Cu^+^	Cu^0^	Cu^+^	Cu^0^		
Cu/SiO_2_	913.8	918.3	1846.3	1850.8	932.5	59.5
0.25B-Cu/SiO_2_	913.8	918.4	1846.4	1851.0	932.6	60.4
1B-Cu/SiO_2_	914.2	918.5	1846.7	1851.0	932.5	62.7
2B-Cu/SiO_2_	914.2	918.5	1846.8	1851.1	932.6	64.6
3B-Cu/SiO_2_	914.4	918.6	1846.8	1851.2	932.6	69.2

^a^ Kinetic energy. ^b^ Auger parameter. ^c^ Intensity ratio between Cu^+^ and (Cu^+^ + Cu^0^) by deconvolution of Cu LMM XAES.

## Data Availability

The data presented in this study are available on request from the corresponding author.

## Supplementary Materials

The following are available online at https://www.mdpi.com/article/10.3390/nano11123236/s1, Figure S1: Schematic diagram of the reaction equipment for DMO hydrogenation to EG and ethanol; Figure S2: N_2_ adsorption–desorption isotherms of calcined *x*B-Cu/SiO_2_ catalysts; Figure S3: EDX image of reduced 2B-Cu/SiO_2_ catalyst; Figure S4: Long-term stability test of 2B-Cu/SiO_2_ catalysts. Reaction conditions: LHSV = 0.2 h^−1^, T=170 °C, P = 2.0 MPa, H_2_/DMO molar ratio = 50; Figure S5: Cu LMM XAES spectra of used catalysts; Table S1: Results of measurements and fitting data for H_2_-TPR of the *x*B-Cu/SiO_2_ catalysts; Table S2: Catalytic performance for the hydrogenation of DMO over *x*B-Cu/SiO_2_ catalysts; Table S3: Struture changes of the Cu/SiO_2_ and 2B-Cu/SiO_2_ catalysts after long-term test.
