# Supplementary material for: Boron Modified Bifunctional Cu/SiO2 Catalysts with Enhanced Metal Dispersion and Surface Acid Sites for Selective Hydrogenation of Dimethyl Oxalate to Ethylene Glycol and Ethanol
Source: Nanomaterials (Basel). 2021 Nov 29;11(12):3236. doi: 10.3390/nano11123236 (PMC8706936; doi:10.3390/nano11123236)
Supplement: Supplementary file 1 [file nanomaterials-11-03236-s001.zip › Supplementary File-latest version.pdf]

nanomaterials

Supplementary Materials

Boron modified bifunctional Cu/SiO2 catalysts with enhanced metal dispersion and surface acid sites for selective hydrogenation of dimethyl oxalate to ethylene glycol and ethanol

Deliang Yang [1,*], Runping Ye [2], Ling Lin [3], Rong Guo [3], Peiyu Zhao [1], Yanchao Yin [1], Wei Cheng [1], Wenpeng Yuan [1] and Yuangen Yao [3,*]

[1] Biological Engineering Technology Innovation Center of Shandong Province, Heze Branch of Qilu University of Technology (Shandong Academy of Sciences), Heze 274000, China; peiyuz0512@126.com (P.Z.); yycbm@163.com (Y.Y.); chengwei200300@163.com (W.C.); yuanwp@sdas.org (W.Y.)
[2] Key Laboratory of Jiangxi Province for Environment and Energy Catalysis, Institute of Applied Chemistry, College of Chemistry, Nanchang University, Nanchang 330031, China; rye@ncu.edu.cn
[3] Key Laboratory of Coal to Ethylene Glycol and Its Related Technology, Fujian Institute of Research on the Structure of Matter, Chinese Academy of Sciences, Fuzhou 350002, China; llin@fjirsm.ac.cn (L.L.); rongg@fjirsm.ac.cn (R.G.)
* Correspondence: sunyunqi_0539@qlu.edu.cn (D.Y.); yyg@fjirsm.ac.cn (Y.Y.); Tel.: +86-178-6503-7000 (D.Y.)

Catalyst characterization

Inductively coupled plasma optical emission spectroscopy (ICP-OES) was used to measure all the metal (Cu, B and Si) elements content of the *x*B-Cu/SiO_2 catalysts on a Jobin Yvon Ultima2 instrument. Textural properties (S_{BET}, V_p and D_p) of the *x*B-Cu/SiO_2 catalysts were collected by means of the Brunauer-Emmett-Teller (BET) and the Barrett-Joyner-Halenda (BJH) methods on a Micromeritics Tristar 3020 instrument at -196 °C.

X-ray diffraction (XRD) and transmission electron microscope (TEM) techniques were used to analyze the phase structure of the *x*B-Cu/SiO_2 catalysts on Rigaku MiniFlexII diffractometer (40 kV tube voltage) and Tecnai F20 apparatus (200 kV acceleration voltage), respectively. The scanning speed of the Cu $K\alpha$ radiation was 5°/min from 2θ = 10° to 80°. The TEM test samples were on the surface of the carbon-coated Cu grids, by means of 30 min of ultrasonic vibration in 25 °C ethanol solvent.

To test the reducibility of the catalyst samples, the H_2-Temperature-programmed reduction (H_2-TPR) test was operated on a Micromeritics Autochem II 2920 analyzer. The catalyst samples were pretreated in an Ar flow for 2 h at 120 °C. After natural cooling to 50 °C, the temperature was raised up to 800 °C with a ramping rate of 10 °C/min under 10 vol.% H_2-Ar. D_{Cu} and S_{Cu} were colleted by N_2O titration method on a Micromeritics Autochem II 2920 analyzer. Specifically, 100 mg of the pretreated samples were reduced under 10 vol.% H_2-Ar for 4 h at 300 °C. And then the reduced catalyst samples were subjected to N_2O for 2 h at 60 °C, which assured that all the surface Cu atoms were oxidized from Cu^0 to Cu^+. After purging the remaining N_2O with Ar flow for 30 min, the catalyst samples were reduced again by pulse titration with high-purity H_2 at 350 °C.

NH_3 temperature-programmed desorption (NH_3-TPD) test was adopted to investigate the acidic of the samples on a TP-5080 instrument. Briefly, 0.1 g of the catalyst was activated in 99.99% H_2 flow at 350 °C for 2 h. After natural cooling to 50 °C, the 99.99% H_2 was switched to 5% NH_3-95% Ar for 1 h. To be continue, the high pure He was used to sweep the excess NH_3, then the temperature was raised up to 800 °C with a ramping rate of 10 °C/min^{-1}.

X-ray photoelectron spectroscopy (XPS) and X-ray induced Auger electron spectroscopy (XAES) measurements were used to identify the surface composition and chemical state of catalysts, which were carried out on an ESCALAB 250Xi (hν = 1486.6 eV).

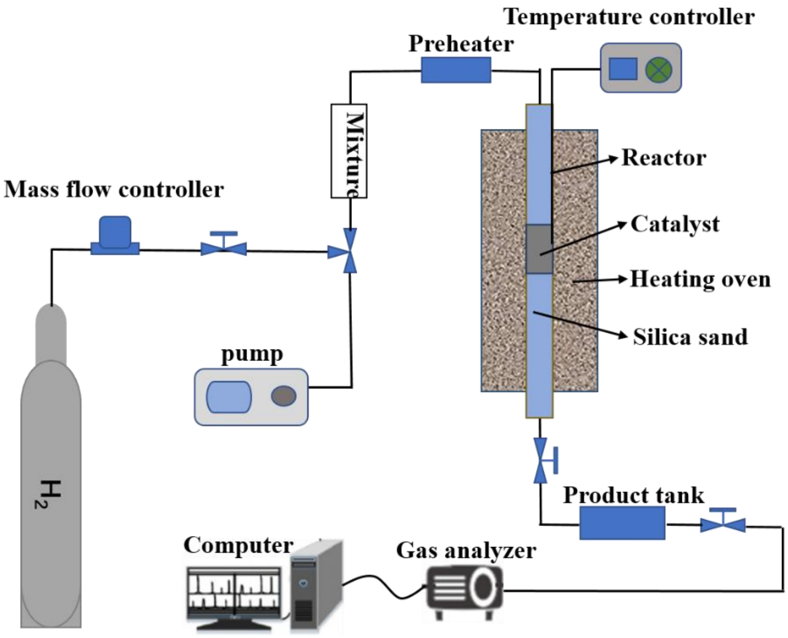

Figure S1. Schematic diagram of the reaction equipment for DMO hydrogenation to EG and EtOH.

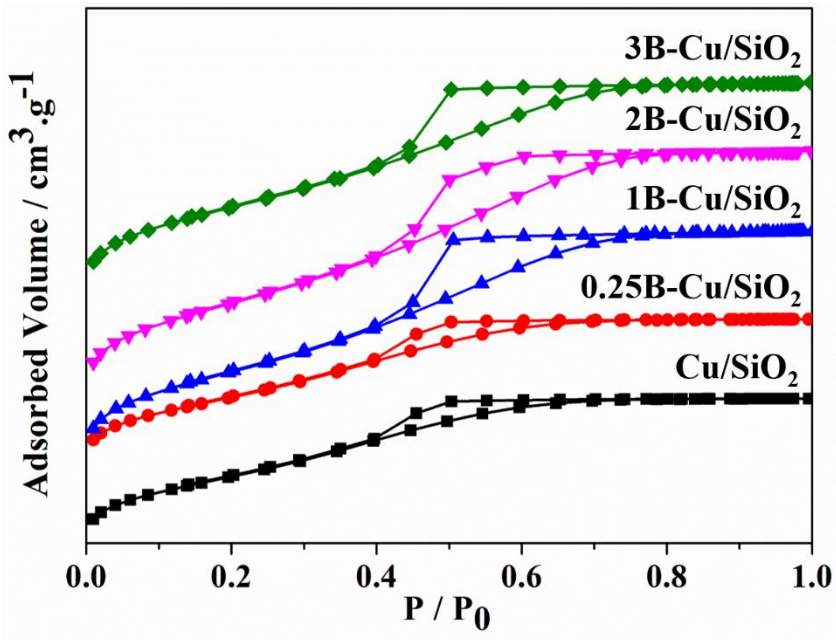

Figure S2. N_2 adsorption–desorption isotherms of calcined *x*B-Cu/SiO_2 catalysts.

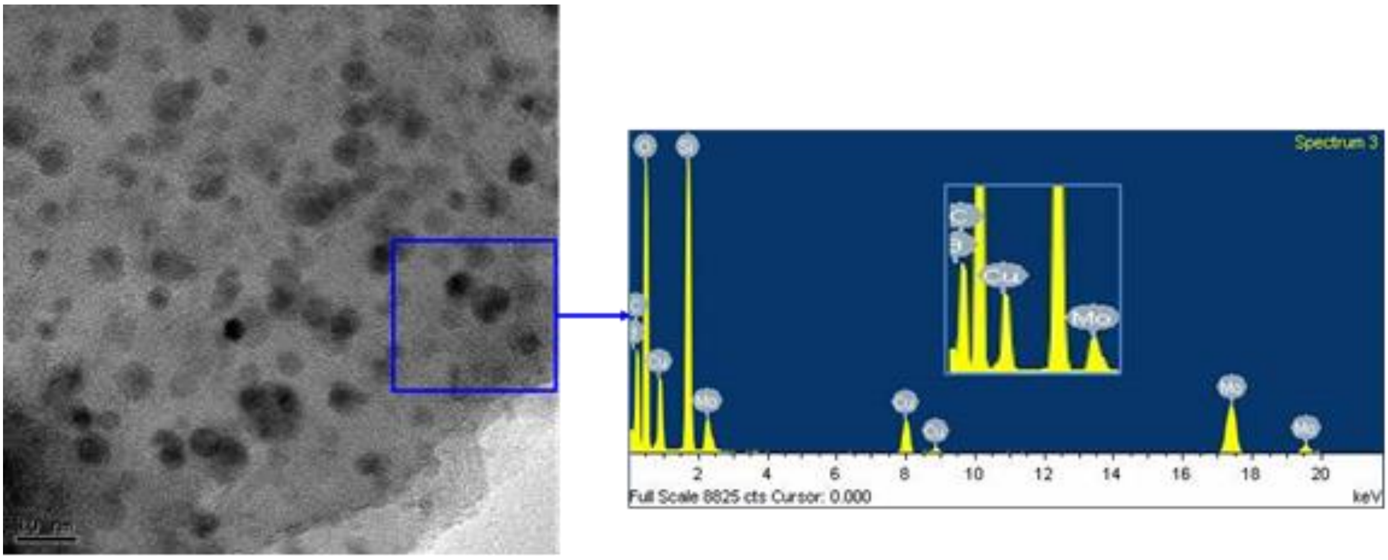

Figure S3. EDS image of reduced 2B-Cu/SiO_2 catalyst.

Table S1. Results of measurements and fitting data for H_2 -TPR of the *x*B-Cu/SiO_2 catalysts.

**Catalysts**	**Total H_2 consumption (mmol·g^{-1})**	**Total theoretical H_2 consumption (mmol·g^{-1})**
Cu/SiO_2	18.9	22.6
0.25B-Cu/SiO_2	16.5	22.4
1B-Cu/SiO_2	18.3	22.1
2B-Cu/SiO_2	17.8	22.0
3B-Cu/SiO_2	17.1	21.9

Table S2. Catalytic performance for the hydrogenation of DMO over *x*B-Cu/SiO_2 catalysts[a].

**Catalyst**	**DMO Con. (%)**	Sel. (%)				TOF[c]
		EG	MG	EtOH	Others[b]	
**Cu/SiO_2**	**30.1**	8.5	91.3	0	0.1	2.5
0.25B- Cu/SiO_2	32.3	8.9	90.9	0	0.1	2.6
1B- Cu/SiO_2	40.6	10.8	88.9	0.1	0.1	3.0
2B- Cu/SiO_2	48.2	13.1	86.2	0.1	0.1	3.4
3B- Cu/SiO_2	42.7	11.6	87.9	0.2	0.2	3.2

[a] Reaction conditions: T = 160 ℃, LHSV = 0.2 h^{-1}, P = 2.0 MPa, H_2/DMO molar ratio = 50.
[b] Others byproducts including 1,2-BDO and 1,2-PDO.
[c] TOF values were calculated by the Cu dispersion of N_2O titration and based on the conversion of DMO in 160 ℃.

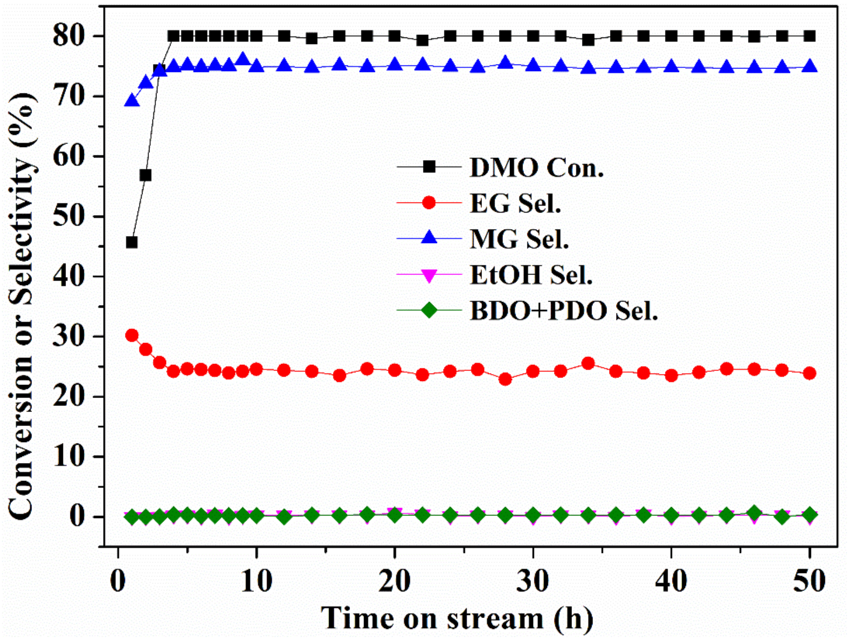

Figure S4. Long-term stability test of 2B-Cu/SiO_2 catalysts. Reaction conditions: LHSV = 0.2 h^{-1}, T=170 ℃, P = 2.0 MPa, H_2/DMO molar ratio = 50.

The procedure to calculate number of acid sites:

n (acidic sites) = n (NH_3 desorbed)/S_{BET}

n (acidic sites): the Density of acidic sites (μmol/m^2)

n (NH_3 desorbed): Total NH_3 desorbed on the surface of 1g catalyst (μmol/g), which is replaced by the total NH_3 desorption peak area simplistically;

S_{BET}: the BET surface area of 1g catalyst determined by N_2 adsorption–desorption isotherms (m^2/g).

The TOF calculation method：

$$TOF = \frac{n\ (DMO\ con.)}{n\ (Cu) * t}$$

n (DMO con.) = $V_{DMO} * C_{DMO} * C'_{DMO} * t$

n (Cu) = $m_{cat.} * n'(Cu)$

t: 1 h

n (DMO con.): the amount of converted DMO molecules

n (Cu): the amount of Cu sites on the surface calculated by N_2O titration

n′(Cu): the numbers of Cu sites on the surface of 1g catalyst calculated by N_2O titration

$m_{cat.}$: the weight of catalyst.

V_{DMO}： the flow of DMO methanol solution per hour by pump

C_{DMO}: the DMO concentration of DMO methanol solution

C'_{DMO}: the conversion of DMO

Table S3. Struture changes of the Cu/SiO_2 and 2B-Cu/SiO_2 catalysts after long-term test.

Catalysts	Cu dispersion (%)[a]	X_{Cu^+} (%)[b]
fresh Cu/SiO_2	17.9	59.5
used Cu/SiO_2	12.5	48.9
fresh 2B-Cu/SiO_2	21.6	64.6
used 2B-Cu/SiO_2	20.4	62.7

[a] Cu metallic surface area determined by N_2O titration method.

[b] Intensity ratio between Cu^+ and (Cu^+ + Cu^0) by deconvolution of Cu LMM XAES.

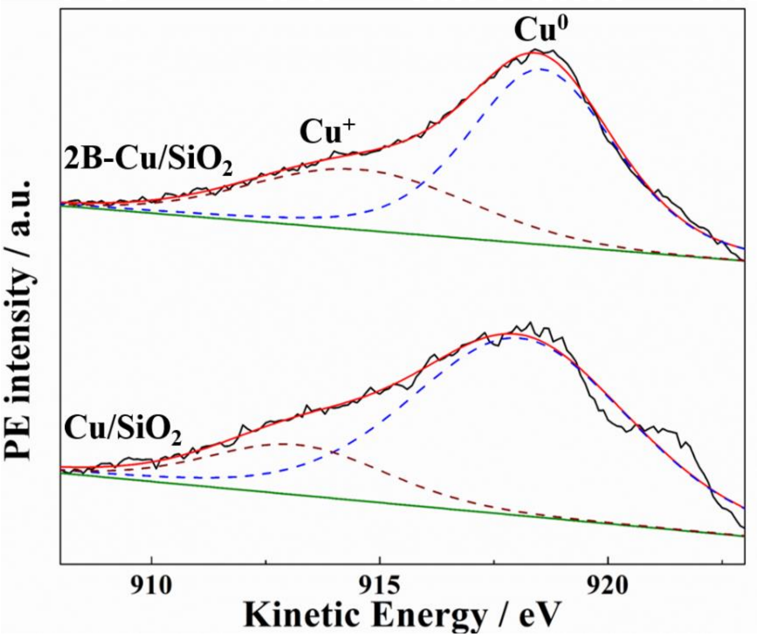

Figure S5. Cu LMM XAES spectra of used catalysts.
